# Association of LPP and TAGAP Polymorphisms with Celiac Disease Risk: A Meta-Analysis

**DOI:** 10.3390/ijerph14020171

**Published:** 2017-02-10

**Authors:** Shi-Qi Huang, Na Zhang, Zi-Xing Zhou, Chui-Can Huang, Cheng-Li Zeng, Di Xiao, Cong-Cong Guo, Ya-Jing Han, Xiao-Hong Ye, Xing-Guang Ye, Mei-Ling Ou, Bao-Huan Zhang, Yang Liu, Eddy Y. Zeng, Guang Yang, Chun-Xia Jing

**Affiliations:** 1 Department of Epidemiology, School of Basic Medical Sciences, Jinan University, No. 601 Huangpu Road West, Guangzhou 510632, Guangdong, China; cookiehaha@sina.cn (S.-Q.H.); lilyronna@outlook.com (N.Z.); m13682215478@163.com (Z.-X.Z.); huangcc531@163.com (C.-C.H.); chengleezeng@163.com (C.-L.Z.); kwstljh@163.com (D.X.); guo301@aliyun.com (C.-C.G.); yajinghan001@outlook.com (Y.-J.H.); 15521143958@163.com (X.-H.Y.); yestar1989@163.com (X.-G.Y.); oumeiling1214@outlook.com (M.-L.O.); candy2006520@163.com (B.-H.Z.); liuyang199101@126.com (Y.L.); 2 Department of Preventive Medicine, Zunyi Medical College, Zhuhai Campus, Zhuhai 519041, Guangdong, China; 3 School of Environment, Guangzhou Key Laboratory of Environmental Exposure and Health, Guangdong Key Laboratory of Environmental Pollution and Health, Jinan University, Guangzhou 510632, Guangdong, China; eddyzeng@jnu.edu.cn; 4 Department of Parasitology, School of Basic Medical Sciences, Jinan University, Guangzhou 510632, Guangdong, China

**Keywords:** LPP, TAGAP, polymorphism, celiac disease, meta-analysis

## Abstract

*Background:* Lipoma preferred partner (*LPP*) and T-cell activation Rho GTPase activating protein (*TAGAP*) polymorphisms might influence the susceptibility to celiac disease. Therefore, we performed a meta-analysis by identifying relevant studies to estimate the risks of these polymorphisms on celiac disease. *Methods:* The PubMed, Web of Science and Embase databases were searched (up to October 2016) for *LPP* rs1464510 and *TAGAP* rs1738074 polymorphisms. *Results:* This meta-analysis included the same 7 studies for *LPP* rs1464510 and *TAGAP* rs1738074. The minor risk A allele at both rs1464510 and rs1738074 carried risks (odds ratios) of 1.26 (95% CI: 1.22–1.30) and 1.17 (95% CI: 1.14–1.21), respectively, which contributed to increased risks in all celiac disease patients by 10.72% and 6.59%, respectively. The estimated lambdas were 0.512 and 0.496, respectively, suggesting that a co-dominant model would be suitable for both gene effects. *Conclusions:* This meta-analysis provides robust estimates that polymorphisms in *LPP* and *TAGAP* genes are potential risk factors for celiac disease in European and American. Prospective studies and more genome-wide association studies (GWAS) are needed to confirm these findings, and some corresponding molecular biology experiments should be carried out to clarify the pathogenic mechanisms of celiac disease.

## 1. Introduction

Celiac disease (CD) is a chronic and immune-mediated enteropathy that is induced by dietary protein gluten (from wheat, barley and rye) in genetically predisposed individuals [1]. It is a small-intestine disorder, affecting approximately 1% of the European population with some regional variations [2] and causing malnutrition and severe complications. Celiac patients have a greater burden of disease than the general population, and a long-term gluten-free diet (GFD) is the only therapy for this disease [1,3]. HLA-DQ2 and HLA-DQ8 molecules are responsible for only approximately 40% of genetic predisposing factors in the pathogenesis of CD [4], which is necessary but not sufficient to cause disease [5,6]. Thus, many more risk loci outside the HLA region should be identified as disease markers.

In recent years, genome-wide association studies (GWAS) have expanded our understanding of genetic makeup and revealed several possible inherited risk factors in celiac disorders [7,8,9,10]. Many of the non-HLA loci overlap with Crohn’s disease, type 1 diabetes, rheumatoid arthritis and juvenile idiopathic arthritis [11,12,13,14,15], such as lipoma preferred partner (*LPP*) and T-cell activation Rho GTPase activating protein (*TAGAP*). Alterations of the actin cytoskeleton and cell shape can be observed in the CD patients’ intestinal mucosa [16,17], while the cell shape is maintained through the actin cytoskeleton and focal adhesion [18]. *LPP* is localized with paxillin in focal adhesions, and the number of paxillin focal adhesions with *LPP* is increased in CD fibroblasts. A constitutive alteration in cell shape and adhesion involving *LPP* occurs in CD fibroblasts, suggesting a correlation between *LPP* and CD pathogenesis [19]. In addition, *LPP* is considered a substrate of the protein-tyrosine-phosphatase 1B (PTP1B) [20]. Of note, loss of PTP1B can attenuate the activation of extracellular signal regulated kinase (ERK) [21], which is activated in the CD patients’ mucosa on a GFD or a gluten-containing diet (GCD). Only when ERK is phosphorylated can it transduce to the nuclei, and it has been found that more nuclei of the enterocytes from CD patients were positive for ERK compared with controls. Inhibition of ERK phosphorylation normalizes crypt enterocyte proliferation of CD atrophic mucosa [22]. When PTP1B is sufficient or excessive, there may be more ERK activity in the celiac enterocytes, resulting in the progression of CD.

*TAGAP* is involved in the Rho GTPase cycle [23,24], which is between the inactive GDP-bound and the active GTP-bound states. The exchange of GDP-bound for GTP-bound is catalyzed by GEFs, while GAPs increase the intrinsic GTPase activity of Rho GTPases to accelerate the return of the proteins to the inactive state [25,26,27]. In the active state, GEF-catalyzed activation of Rho interacts with ROCK, which can activate the myosin light chain (MLC) and LIM domain kinase (LIMK), and both of them play an important role in focal adhesion and regulate the rearrangement and stabilization of the actin cytoskeleton [28]. However, *TAGAP* propagates the inactive form of the RHO molecule; and it increases the activity of Rho GTPases via phosphorylation, enhancing their intrinsic activity up to fivefold [29]. *TAGAP* negatively regulates downstream effects; thus, the actin cytoskeleton rearrangement is dysfunctional and lack of unstable [23].

Mutation of *LPP* and *TAGAP* may interfere with their original function and even promote the progress of CD. In recent years, a number of studies, including GWAS, have reported the association of *LPP* and *TAGAP* polymorphisms with CD susceptibility, and many have focused on *LPP* rs1464510 (A/C) and *TAGAP* rs1738074 (A/G). However, those studies have drawn inconsistent conclusions due to the limited regions and small numbers of articles. For example, Dubois et al. [8] reported that rs1464510 was positively associated with CD in the Netherlands, whereas there was no relationship in a Dutch population according to Coenen et al. [30] and Hunt et al. [9]. Similarly, results for rs1738074 differed from country to country in the studies by Plaza-Izurieta et al. [7] and Sperandeo et al. [31]. Therefore, we decided to carry out this meta-analysis on all the available case-control studies to accurately assess the relationship between the *LPP* rs1464510/*TAGAP* rs1738074 and CD risk.

## 2. Materials and Methods

### 2.1. Search Strategy

Relevant studies were searched in PubMed, the Web of Science and Embase up to October 2016. The search strategies were as followed: (((*LPP* or 3q28 or rs1464510 or “lipoma preferred partner”) or “lim domain containing preferred translocation protein”) and celiac disease) or ((*TAGAP* or 6q25 or rs1738074 or “T-cell activation GTPase activating protein”) and celiac disease). The search was limited to English-language and human studies. Only published studies were considered. We scanned the title and abstract of all relevant articles, manually examined reference lists for additional relevant publications and obtained the full text of all possibly relevant studies. If multiple articles were published on the same topic, the most complete and recent study was used.

### 2.2. Inclusion and Exclusion Criteria

A reviewer independently examined the titles and abstracts of the identified articles. Any human population-based association study was included regardless of subjects’ ethnicity if it met the following criteria: (1) it showed an association between *LPP* (rs1464510) or *TAGAP* (rs1738074) polymorphism, (2) the outcome was celiac disease and there was a control group, (3) there were sufficient data for extraction (i.e., minor allele frequency and genotype frequency) and (4) there was a clear diagnosis of celiac disease. Studies were excluded if: (1) the case and control subjects were biologically related; (2) the insufficient data that were failed to ask for supplementary information from the authors; (3) the studies comprised unrelated data, family studies, animal studies, reviews, or meeting abstracts; or (4) the studies were written not in English.

### 2.3. Data Extraction

Summary data were extracted independently by reviewers using a standardized data extraction form. We extracted general information as follows: name of first author, year of publication, region of study population, source of controls, genotype method, diagnostic criteria, the number of cases and controls, and the minor allele frequency in cases and controls. Any disagreement was resolved by consensus.

### 2.4. Risk of Bias Assessment

Study quality was assessed independently by the same reviewers using a risk-of-bias score for genetic association studies that was developed by Thakkinstian et al. [32] (Supplementary Materials Table S1). The score considered 5 domains: information bias (ascertainment of outcome and gene), confounding bias, selective reporting of outcomes, population stratification, and Hardy-Weinberg equilibrium (HWE) assessment in the control group. Each item was scored “yes”, “no” or “unclear”, representing low risk, high risk and insufficient information, respectively. Disagreement between the two reviewers was solved by a senior reviewer (C.X.J). Additionally, the MOOSE checklist was used to measure the quality of our study (Supplementary Materials Table S2).

### 2.5. Statistical Analysis

We used Stata software (version 12.0, StataCorp LLC, College Statopm, TX, USA) and the Comprehensive Meta-Analysis software (version 2.0, Biostat, Englewood, NJ, USA) for all statistical analyses. All tests with a *p* value less than 0.05 were considered statistically significant, except for the heterogeneity tests, in which a *p* value less than 0.10 was used. It was tested whether the distribution of genotypes in the controls was compliant with Hardy-Weinberg equilibrium (HWE) by a Fisher’s exact test to estimate the quality of studies. If the study was found not to be in HWE with a *p* value less than 0.05, it was considered to be in disequilibrium. We used both per-allele and per-genotype analysis to estimate the strength of the association between the polymorphism of *LPP* rs1464510 or *TAGAP* rs1738074 and CD risks.

Per-allele analysis: Suppose that A and a are risk and non-risk alleles, respectively, and AA, Aa and aa are minor homozygous, heterozygous, and common homozygous genotypes, respectively, for each polymorphism. The risk allele frequency in each group was estimated according to the reported genotype data, and overall prevalence along with 95% confidence intervals were estimated for each single nucleotide polymorphism (SNP). The Mantel-Haenszel method was used to determine the statistical significance of the pooled OR, and its *p* value was used to determine whether the overall gene effect was significant (*p* = 0.05). The heterogeneity of allele effects across studies was checked using a Q test, and the degree of heterogeneity was quantified by I^2^ (I^2^ < 25%, no heterogeneity; 25% < I^2^ < 50%, moderate heterogeneity; 50% < I^2^ < 75%, large heterogeneity; I^2^ > 75%, extreme heterogeneity). If heterogeneity was present (i.e., if the Q test was significant or I^2^ was greater than 25%), the cause of heterogeneity was explored using sensitivity analysis. We chose a random-effects model if I^2^ was greater than 50%; otherwise, a fixed-effects model was used. The population attributable risk (PAR) for the risk allele was calculated based on results from a discrete-time model. If the main effect of the genotype was statistically significant and had the appropriate effect model selection, further comparisons of OR_1_ and OR_2_ were explored. Per-genotype analysis: We used the model-free approach to estimate the genotype effect, and two odds ratios—AA vs. aa (OR_1_) and Aa vs. aa (OR_2_)—were estimated for each study. The model of the genetic effect, measured by the parameter lambda (λ), which is defined as the ratio of logOR_2_ to logOR_1_, was then estimated using the model-free Bayesian approach. Lambda (λ) represents the heterozygote effect as a proportion of the homozygote variant effect. The value of lambda ranges from 0 to 1. We obtained information about the genetic mode of action as follows: If λ = 0, a recessive (Aa + aa vs. AA) model is suggested; if λ = 1, a dominant model (AA + Aa vs. aa) is suggested; and if λ = 0.5, a co-dominant model (AA vs. aa, Aa vs. aa) is suggested. If λ > 1 or λ < 0, then a homozygous or heterosis model is likely, although this is rare. Once the best genetic model is identified, this model is used to collapse the three genotypes into two groups and to pool the results again. For lambda, WinBugs 1.4.2 was used with vague prior to distributions for the estimation of parameters (i.e., lambda and odds ratio). The models were run with a burn-in of 1000 iterations, followed by 10,000 iterations for parameter estimates. The Begg and Mazuma rank correlation and Egger’s test were adopted to assess and quantify the publication bias. A sensitivity analysis was performed, and we removed studies one by one to reflect the influence of each study on the pooled OR of the others. In addition, we calculated the classic fail-safe N value using Comprehensive Meta-Analysis software (version 2.0) to quantitatively evaluate the reliability of the results.

## 3. Results

### 3.1. Identifying Relevant Studies

**Figure 1:**
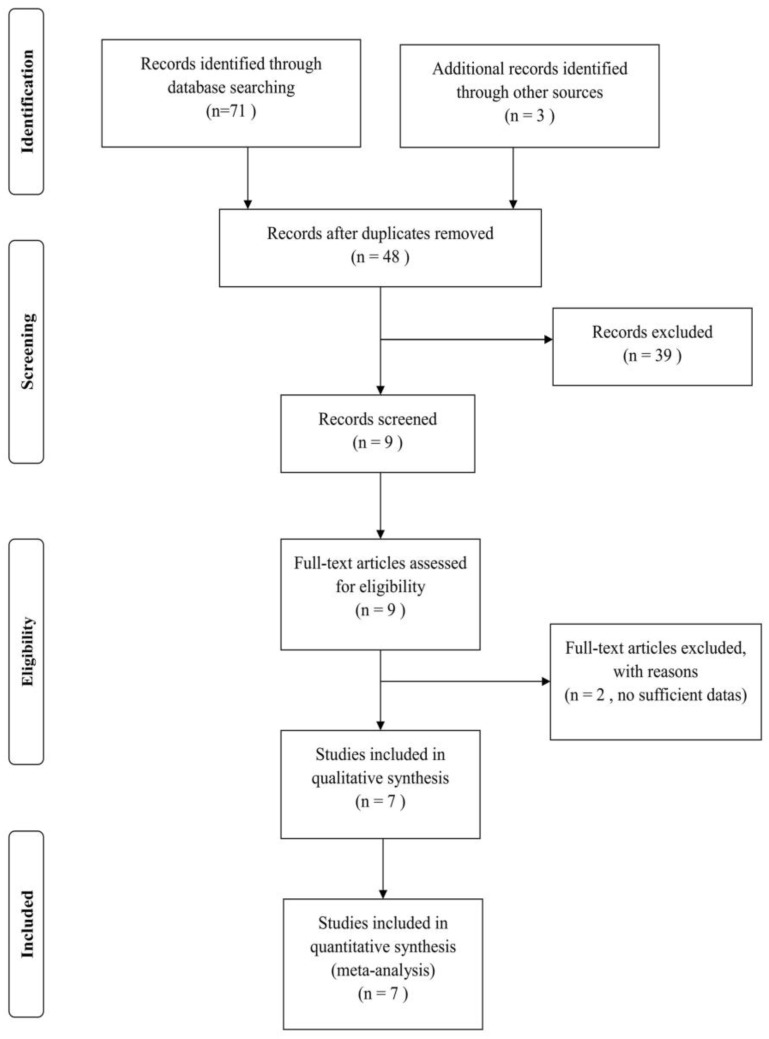
Flow chart for identified studies for *LPP* and *TAGAP* genes with CD.

Twenty-five, twenty-one and twenty-five studies were identified from PubMed, Web of Science, and Embase, respectively; an additional three studies were identified from references in the included studies (Figure 1). After duplicates were removed, there were forty-eight studies, thirty-nine of which were ineligible. The ineligible records consisted of seventeen other studies, one animal study, three review articles, three family studies, six meeting articles, one meta-analysis of inflammatory bowel disease, two studies without the target SNPs, and six studies aimed at other immune diseases. After retrieving and reviewing the nine remaining studies, we excluded two studies without sufficient data, leaving seven studies to be used for further data extractions (Table 1).

### 3.2. Risk of Bias Assessment

The results of bias assessment are presented in Table 2. Each study was compliant with HWE. All studies had a low risk of bias from population stratification, selective outcome reports, ascertainment of celiac disease and ascertainment of control. The risk of bias was highest in quality control for genotyping and confounding bias (both unclear in 1 study, 14.29%).

### 3.3. Association between the LPP rs1464510 Polymorphism and CD Risk

**Figure 2:**
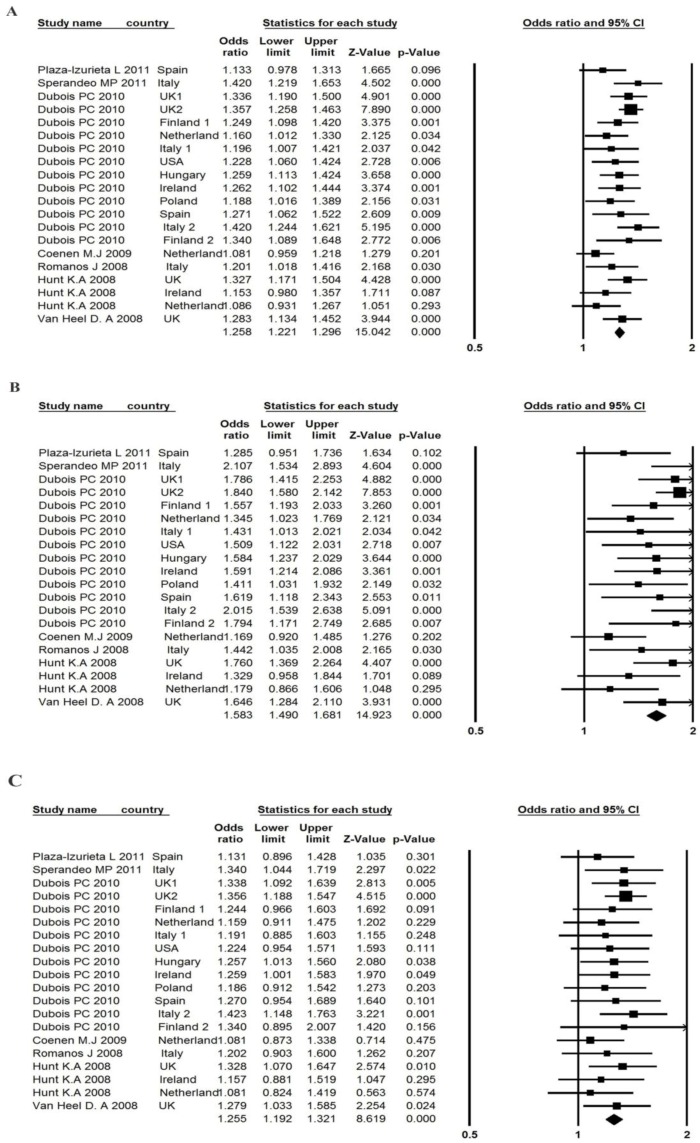
Forest plot of the association between *LPP* rs1464510 polymorphism and CD risk in (**A**) A vs. C; (**B**) AA vs.CC; (**C**) AC vs. CC.

The seven studies reported an association between *LPP* rs1464510 polymorphism and CD, with 14,936 cases and 24,788 controls (Table 3). The pooled OR (A vs. C) showed moderate heterogeneity (*p* = 0.106, and I^2^ = 29.52%) across the studies, with a pooled OR of 1.26 (95% CI: 1.22, 1.30) (part A of Figure 2), suggesting that individuals carrying the risk A allele had a 26% higher risk of developing CD than those carrying the C allele. The PAR for risk allele A was 10.72%. The sensitivity analysis suggested that, if we excluded the study by Coenen et al. [30], I^2^ was reduced from 29.52% to 11.64% and the pooled odds ratio was 1.27 (95% CI: 1.23, 1.31) (Supplementary Materials Table S3). The Egger test (*p* = 0.100) and Begg and Mazumdar rank correlation (*p* = 0.284) suggested that no publication bias existed. Publication bias was also tested using a funnel plot (Supplementary Materials Figure S1). The classic fail-safe N value was 1032 (Z = 14.21; *p* = 0.00), which suggested that 1032 unpublished negative studies would have to be included to convert the combined *p* value to a non-significant value.

The genotype frequency and estimated ORs of LPP rs1464510 are presented in parts B and C of Figure 2. The OR_1_ (AA vs. CC) (*p* = 0.097; I^2^ = 30.45%) was moderately heterogeneous, and the OR_2_ (AC vs. CC) (*p* = 0.979; I^2^ = 0.0%) was homogenous. The pooled OR_1_ (1.58; 95% CI: 1.49, 1.68; *p* < 0.001) and OR_2_ (1.26; 95% CI: 1.19, 1.32; *p* < 0.001) were statistically significant, which indicated that persons with AA and AC genotypes in LPP rs1464510 had an approximately 58% and 26% higher risk, respectively, of developing CD than persons with the CC genotype. The Egger test did not suggest any asymmetry for both ORs (*p* = 0.133 for OR_1_, *p* = 0.054 for OR_2_). The λ was 0.512 (95% CI: 0.388, 0.660), suggesting that a co-dominant effect was most likely.

### 3.4. Association between the TAGAP rs1738074 Polymorphism and CD Risk

**Figure 3:**
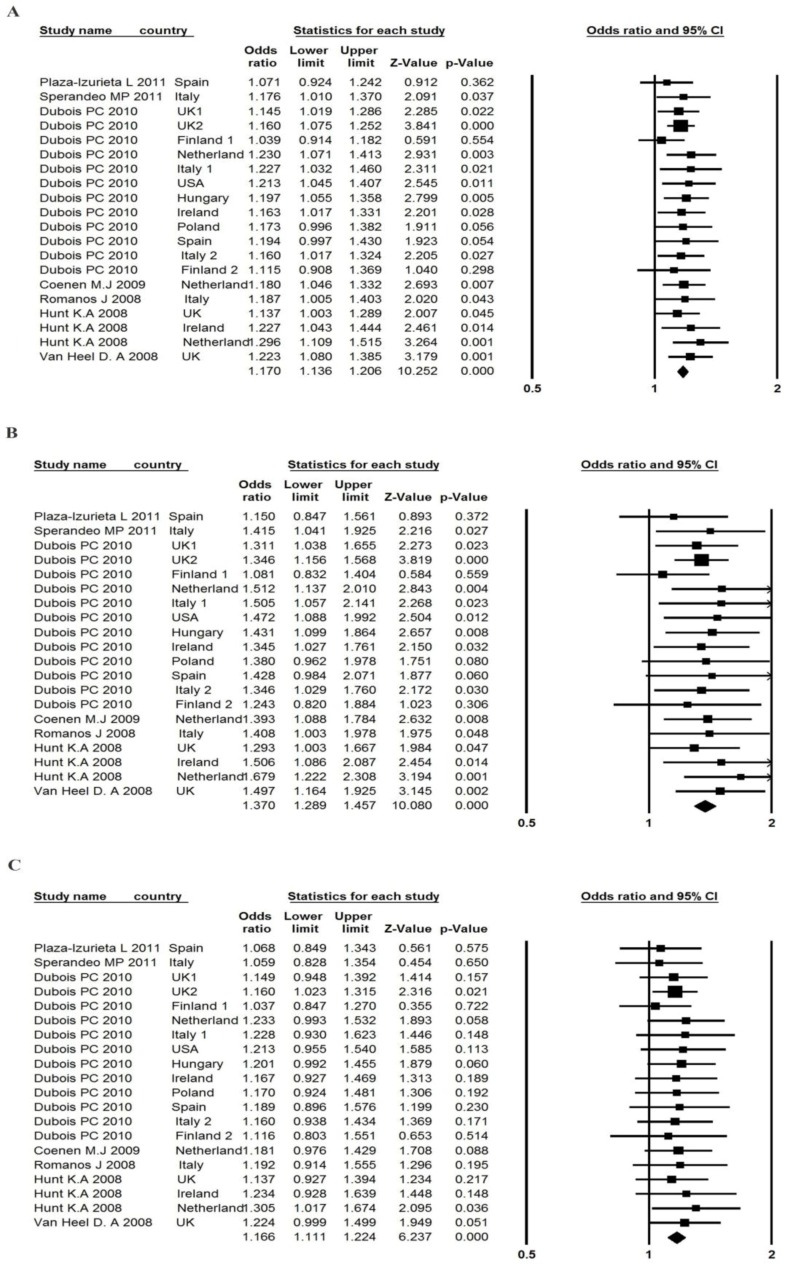
Forest plot of the association between *TAGAP* rs1738074 polymorphism and CD risk in (**A**) A vs. G; (**B**) AA vs.GG; (**C**) AG vs. GG.

The seven studies reported an association between *TAGAP* rs1738074 polymorphism and CD, with 14,936 cases and 24,788 controls (Table 4). The pooled OR (A vs. G) was 1.17 (95% CI: 1.14, 1.21), estimated by the fixed-effects model (*p* = 0.974, and I^2^ = 0.00%) (part A of Figure 3), which suggested that individuals carrying the risk A allele had a 17% higher risk of developing CD than those carrying the G allele. The PAR for risk allele A was 6.59%. The Egger test (*p* = 0.440) and Begg and Mazumdar rank correlation (*p* = 0.315) suggested that no publication bias existed. Publication bias was also tested using a funnel plot (Supplementary Materials Figure S2). The classic fail-safe N value was 513 (Z = 10.11; *p* = 0.00), which suggested that 513 unpublished negative studies would have to be included to convert the combined *p* value to a non-significant value.

The OR_1_ (AA vs. GG, 1.37; 95% CI: 1.29, 1.46; *p* < 0.001) and the OR_2_ (AG vs. GG, 1.17; 95% CI: 1.11, 1.22; *p* < 0.001) were homogenous, and estimated by a fixed-effects model in parts B and C of Figure 3. The results can be interpreted as indicating that persons with AA and AG genotypes in *TAGAP* rs1738074 had approximately 37% and 17% higher risks, respectively, of developing CD than persons with the GG genotype. Egger’s test did not suggest any asymmetry for both ORs (*p* = 0.425 for OR_1_, *p* = 0.611 for OR_2_). The λ was 0.496 (95% CI: 0.310, 0.711), which suggested that a co-dominant effect was most likely.

## 4. Discussion

Our meta-analysis suggests that both *LPP* rs1464510 and *TAGAP* rs1738074 polymorphisms contribute to the susceptibility to CD in European and American.

The pooled OR (A vs. C) of *LPP* suffered from moderate heterogeneity, but I² decreased significantly (from 29.52% to 11.63%) when we eliminated The Netherlands data from Coenen et al. [30], indicating that heterogeneity originated mainly from this study. The results between different studies are often heterogeneous, and there are three feasible reasons for such heterogeneity in genetic association studies: association in one population rather than in another, different studies without comparable measures of phenotype, or deviation from HWE [34]. Therefore, we speculate that the main underlying cause of heterogeneity might be populations of various ethnicities.

*LPP*, which is strongly expressed in the small intestine, participates in the regulation of cell adhesion, cytoskeletal remodeling and maintenance of cell shape and motility [35,36], and it seems to be activated more strongly in biopsy specimens from CD patients than in those from non-CD controls [7]. We infer that mutations in the *LPP* lead to the PTP1B becoming sufficient or even excessive, so more ERK may be activated, and that it may play a functional role in CD enterocyte proliferation. Our results suggested a powerful relationship between CD and the *LPP* of rs1464510 (*p* < 0.001, OR = 1.26, 95% CI: 1.22–1.30).*TAGAP* is a Rho GTPase-activating protein crucial for modulating cytoskeletal changes [9,11,12], and it is thought to be a negative regulator of cell signaling and relevant to the regulation of the Rho GTPase cycle [37]. Therefore, we hypothesize that mutations in the *TAGAP* rs1738074 might increase GTPase activity, which propagates the inactive form of the Rho molecule in the Rho GTPase cycle and leads to negative regulation of downstream effects, thus promoting the development of CD. Our meta-analysis confirmed the involvement of rs1738074 in CD susceptibility (*p* < 0.001, OR = 1.17, 95% CI: 1.14–1.21), so pathway analysis should be implemented to generate hypotheses for clarifying the biological link between *TAGAP* and CD [38].

There are some limitations of our study. First, we only included European (38197/39725) and American (1528/39725) populations; nonetheless, our results provide a comprehensive overview of the association between *LPP* rs1464510/*TAGAP* rs1738074 and CD in European populations. Second, all included studies were case-control studies, which might have overestimated the genetic association; a population-based nested case-control study is needed to avoid this bias. Finally, because only English-language literature was retrieved, we may have missed relevant articles written in other languages.

## 5. Conclusions

In summary, our meta-analysis reveals that both *LPP* rs1464510 and *TAGAP* rs1738074 are associated with CD susceptibility. Furthermore, the gene–gene and gene–environment interactions should be evaluated, and studies with larger and more diverse samples should be performed to confirm the results of this meta-analysis.

## Figures and Tables

**Table 1:** Characteristics of the eligible studies for *LPP* and *TAGAP* in meta-analysis.

Authors, Year (Ref.)	Ethnicity	Genotype Method	Gene	Type of SNP	MAF		Sample Size	
					Case	Control	Case	Control
Plaza-Izurieta et al., 2011 [7]	Spanish	RT-PCR	*LPP*	rs1464510	0.450	0.419	1094	540
			*TAGAP*	rs1738074	0.423	0.406		
Sperandeo et al., 2011 [31]	Italian	TaqMan	*LPP*	rs1464510	0.493	0.406	637	711
			*TAGAP*	rs1738074	0.465	0.425		
Dubois et al., 2010 [8]	British	Illumina Hap300v1-1 + IlluminaHap550-2v3	*LPP*	rs1464510	0.522	0.450	737	2596
			*TAGAP*	rs1738074	0.472	0.438		
	British	Illumina 670-QuadCustom_v1 + Illumina 1.2MDuoCustom_v1	*LPP*	rs1464510	0.524	0.448	1849	4936
			*TAGAP*	rs1738074	0.475	0.438		
	Finnish	Illumina 670-QuadCustom_v1 + Illumina610-Quad	*LPP*	rs1464510	0.601	0.547	647	1829
			*TAGAP*	rs1738074	0.430	0.421		
	Dutch	Illumina 670-QuadCustom_v1	*LPP*	rs1464510	0.531	0.493	803	846
			*TAGAP*	rs1738074	0.445	0.395		
	Italian	Illumina 670-QuadCustom_v1	*LPP*	rs1464510	0.517	0.472	497	543
			*TAGAP*	rs1738074	0.464	0.413		
	American	IlluminaGoldenGate	*LPP*	rs1464510	0.511	0.459	973	555
			*TAGAP*	rs1738074	0.470	0.423		
	Hungarian	IlluminaGoldenGate	*LPP*	rs1464510	0.533	0.475	965	1067
			*TAGAP*	rs1738074	0.415	0.372		
	Irish	IlluminaGoldenGate	*LPP*	rs1464510	0.501	0.443	597	1456
			*TAGAP*	rs1738074	0.500	0.462		
	Polish	IlluminaGoldenGate	*LPP*	rs1464510	0.495	0.452	564	716
			*TAGAP*	rs1738074	0.364	0.328		
	Spanish	IlluminaGoldenGate	*LPP*	rs1464510	0.462	0.403	550	433
			*TAGAP*	rs1738074	0.443	0.400		
	Italian	IlluminaGoldenGate	*LPP*	rs1464510	0.495	0.408	1010	804
			*TAGAP*	rs1738074	0.461	0.425		
	Finnish	IlluminaGoldenGate + Illumina610-Quad	*LPP*	rs1464510	0.602	0.531	259	653
			*TAGAP*	rs1738074	0.448	0.421		
Coenen et al., 2009 [30]	Dutch	Illumina HAP550	*LPP*	rs1464510	0.530	0.510	795	1683
			*TAGAP*	rs1738074	0.440	0.400		
Romanos et al., 2008 [33]	Italian	TaqMan technology	*LPP*	rs1464510	0.520	0.474	538	593
			*TAGAP*	rs1738074	0.454	0.412		
Hunt et al., 2008 [9]	British	IlluminaGoldenGate	*LPP*	rs1464510	0.517	0.446	719	1561
			*TAGAP*	rs1738074	0.460	0.428		
	Irish	IlluminaGoldenGate	*LPP*	rs1464510	0.483	0.448	416	957
			*TAGAP*	rs1738074	0.519	0.468		
	Dutch	IlluminaGoldenGate	*LPP*	rs1464510	0.521	0.500	508	888
			*TAGAP*	rs1738074	0.459	0.395		
Van Heel et al., 2008 [10]	British	Illumina Hap300	*LPP*	rs1464510	0.519	0.457	778	1422
			*TAGAP*	rs1738074	0.472	0.422		

RT-PCR: transcriptase PCR; MAF: Minor allele frequency; SNP: single nucleotide polymorphism; Minor allele in *LPP* rs1464510 is A, and minor allele in *TAGAP* rs1738074 is A.

**Table 2:** The risk of bias assessment.

Author, Year (Ref.)	Ascertainment of Celiac Disease	Ascertainment of Control	Quality Control for Genotyping	Population Stratification	Confounding Bias	Selective Outcome Report	HWE
Plaza-Izurieta et al., 2011 [7]	Yes	Yes	Yes	Yes	Yes	Yes	Yes
Sperandeo et al., 2011 [31]	Yes	Yes	Yes	Yes	Yes	Yes	Yes
Dubois et al., 2010 [8]	Yes	Yes	Yes	Yes	Yes	Yes	Yes
Coenen et al., 2009 [30]	Yes	Yes	Yes	Yes	Yes	Yes	Yes
Romanos et al., 2008 [33]	Yes	Yes	Unclear	Yes	Unclear	Yes	Yes
Hunt et al., 2008 [9]	Yes	Yes	Yes	Yes	Yes	Yes	Yes
Van Heel et al., 2008 [10]	Yes	Yes	Yes	Yes	Yes	Yes	Yes

HWE: Hard-Weinberg Equilibrium.

**Table 3:** Genotype frequencies for *LPP* rs1464510and genotype effects of studies included in the meta-analysis.

Author (Ref.)	Country	Case Genotype			Control Genotype			A vs. C		AA vs. CC		AC vs. CC		HWE
		AA	AC	CC	AA	AC	CC	OR	95% CI	OR	95% CI	OR	95% CI	
Plaza-Izurieta et al. [7]	Spain	222	541	331	95	263	182	1.133	0.978–1.313	1.258	0.951–1.736	1.131	0.896–1.428	0.999
Sperandeo et al. [31]	Italy	152	324	161	108	362	241	1.420	1.219–1.653	2.107	1.534–2.893	1.340	1.044–1.719	0.141
Dubois et al. [8]	UK1	201	368	168	526	1285	785	1.336	1.190–1.500	1.786	1.415–2.253	1.338	1.092–1.639	0.997
	UK2	508	922	419	991	2441	1504	1.357	1.258–1.463	1.840	1.580–2.142	1.356	1.188–1.547	0.992
	Finland 1	234	310	103	547	907	375	1.249	1.098–1.420	1.557	1.193–2.033	1.244	0.966–1.603	0.978
	The Netherlands	226	400	177	206	423	217	1.160	1.012–1.330	1.345	1.023–1.769	1.159	0.911–1.475	0.996
	Italy 1	133	248	116	121	271	151	1.196	1.007–1.421	1.431	1.013–2.021	1.191	0.885–1.603	0.977
	USA	254	486	233	117	276	162	1.228	1.060–1.424	1.509	1.122–2.031	1.224	0.954–1.571	0.978
	Hungary	274	480	211	241	532	294	1.259	1.113–1.424	1.584	1.237–2.029	1.257	1.013–1.560	0.991
	Ireland	150	298	149	286	718	452	1.262	1.102–1.444	1.591	1.214–2.086	1.259	1.001–1.583	0.977
	Poland	138	282	144	146	355	215	1.188	1.016–1.389	1.411	1.031–1.932	1.186	0.912–1.542	0.980
	Spain	117	274	159	70	209	154	1.271	1.062–1.522	1.619	1.118–2.343	1.270	0.954–1.689	0.948
	Italy 2	247	505	258	134	388	282	1.420	1.244–1.621	2.015	1.539–2.638	1.423	1.148–1.763	0.978
	Finland 2	94	124	41	184	325	144	1.340	1.089–1.648	1.794	1.171–2.749	1.340	0.895–2.007	0.983
Coenen et al. [30]	The Netherlands	223	396	176	438	841	404	1.081	0.959–1.218	1.169	0.920–1.485	1.081	0.873–1.338	0.994
Romanos et al. [33]	Italy	145	269	124	133	296	164	1.201	1.018–1.416	1.442	1.035–2.008	1.202	0.903–1.600	0.980
Hunt et al. [9]	UK	192	359	168	311	771	479	1.327	1.171–1.504	1.760	1.369–2.264	1.328	1.070–1.647	0.981
	Ireland	97	208	111	192	473	292	1.153	0.980–1.357	1.329	0.958–1.844	1.157	0.881–1.519	0.986
	The Netherlands	138	253	117	222	444	222	1.086	0.931–1.267	1.179	0.866–1.606	1.081	0.824–1.419	1.000
Van Heel et al. [10]	UK	210	388	180	297	706	419	1.283	1.134–1.452	1.646	1.284–2.110	1.279	1.033–1.585	0.990
Overall odds ratio	-	-	-	-	-	-	-	1.258	1.221–1.296	1.583	1.490–1.681	1.255	1.192–1.321	-

**Table 4:** Genotype frequencies for *TAGAP* rs1738074 and genotype effects of studies included in the meta-analysis.

Author (Ref.)	Country	Case Genotype			Control Genotype			A vs. G		AA vs. GG		AG vs. GG		HWE
		AA	AG	GG	AA	AG	GG	OR	95% CI	OR	95% CI	OR	95% CI	
Plaza-Izurieta et al. [7]	Spain	196	534	364	89	261	190	1.071	0.924–1.242	1.150	0.847–1.561	1.068	0.849–1.343	0.968
Sperandeo et al. [31]	Italy	144	305	188	125	354	231	1.176	1.010–1.370	1.415	1.041–1.925	1.059	0.828–1.354	0.596
Dubois et al. [8]	UK1	164	367	205	498	1278	820	1.145	1.019–1.286	1.311	1.038–1.655	1.149	0.948–1.392	0.999
	UK2	417	922	510	947	2430	1559	1.160	1.075–1.252	1.346	1.156–1.568	1.160	1.023–1.315	0.999
	Finland 1	120	317	210	324	892	613	1.039	0.914–1.182	1.081	0.832–1.404	1.037	0.847–1.270	0.987
	The Netherlands	159	397	247	132	404	310	1.230	1.071–1.413	1.512	1.137-2.010	1.233	0.993–1.532	0.984
	Italy 1	107	247	143	93	263	187	1.227	1.032–1.460	1.505	1.057–2.141	1.228	0.930–1.623	0.974
	USA	215	485	273	99	271	185	1.213	1.045–1.407	1.472	1.088–1.992	1.213	0.955–1.540	0.989
	Hungary	166	469	330	148	498	421	1.197	1.055–1.358	1.431	1.099–1.864	1.201	0.992–1.455	0.970
	Ireland	149	299	149	311	724	421	1.163	1.017–1.331	1.345	1.027–1.761	1.167	0.927–1.469	0.993
	Poland	75	261	228	77	316	323	1.173	0.996–1.382	1.380	0.962–1.978	1.170	0.924–1.481	0.982
	Spain	108	271	171	69	208	156	1.194	0.997–1.430	1.428	0.984–2.071	1.189	0.896–1.576	0.981
	Italy 2	215	502	293	145	393	266	1.160	1.017–1.324	1.346	1.029–1.760	1.160	0.938–1.434	0.994
	Finland 2	52	128	79	116	318	219	1.115	0.908–1.369	1.243	0.820–1.884	1.116	0.803–1.551	0.976
Coenen et al. [30]	The Netherlands	154	392	249	269	808	606	1.180	1.046–1.332	1.393	1.088–1.784	1.181	0.976–1.429	0.990
Romanos et al. [33]	Italy	111	267	160	101	287	205	1.187	1.005–1.403	1.408	1.003–1.978	1.192	0.914–1.555	0.974
Hunt et al. [9]	UK	152	357	210	286	764	511	1.137	1.003–1.289	1.293	1.003–1.667	1.137	0.927–1.394	0.988
	Ireland	112	208	96	210	476	271	1.227	1.043–1.444	1.506	1.086–2.087	1.234	0.928–1.639	0.971
	The Netherlands	107	252	148	139	424	325	1.296	1.109–1.515	1.679	1.222–2.308	1.305	1.017–1.674	0.971
Van Heel et al. [10]	UK	173	388	217	253	694	475	1.223	1.080–1.385	1.497	1.164–1.925	1.224	0.999–1.499	0.986
Overall odds ratio	-	-	-	-	-	-	-	1.170	1.136–1.206	1.370	1.289–1.457	1.166	1.111–1.224	-

## Supplementary Materials

The following are available online at www.mdpi.com/1660-4601/14/2/171/s1, Figure S1: Funnel plot for *LPP* rs1464510 (A vs. C) with CD. Figure S2: Funnel plot for *TAGAP* rs1738074 (A vs. G) with CD. Table S1: Risk of bias assessment for genetic association studies of CD of studies included in the meta-analysis. Table S2: MOOSE checklist: The association of *LPP* and *TAGAP* genes with CD risks: a meta-analysis. Table S3: The sensitivity analysis of *LPP* rs1464510 and CD risk (A vs. C).
