# Supplementary material for: Association of LPP and TAGAP Polymorphisms with Celiac Disease Risk: A Meta-Analysis
Source: Int J Environ Res Public Health. 2017 Feb 10;14(2):171. doi: 10.3390/ijerph14020171 (PMC5334725; doi:10.3390/ijerph14020171)
Supplement: Supplementary file 1 [file ijerph-14-00171-s001.pdf]

Association of LPP and TAGAP Polymorphisms with Celiac Disease Risk: A Meta-Analysis

Shi-Qi Huang, Na Zhang, Zi-Xing Zhou, Chui-Can Huang, Cheng-Li Zeng, Di Xiao, Cong-Cong Guo, Ya-Jing Han, Xiao-Hong Ye, Xing-Guang Ye, Mei-Ling Ou, Bao-Huan Zhang, Yang Liu, Eddy Y. Zeng, Guang Yang and Chun-Xia Jing

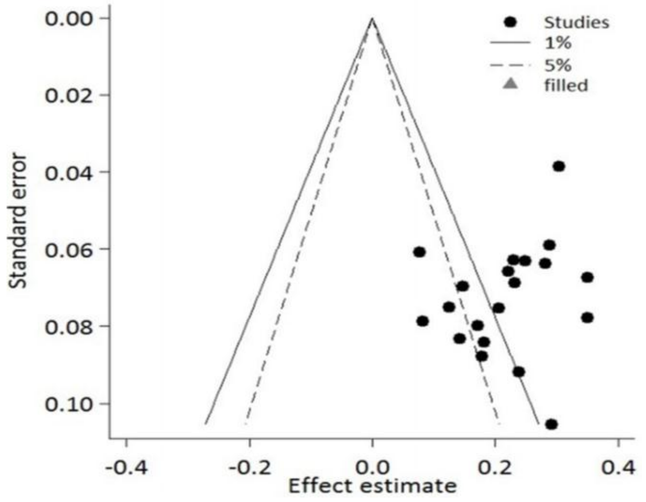

Figure S1. Funnel plot for *LPP* rs1464510 (A vs. C) with CD.

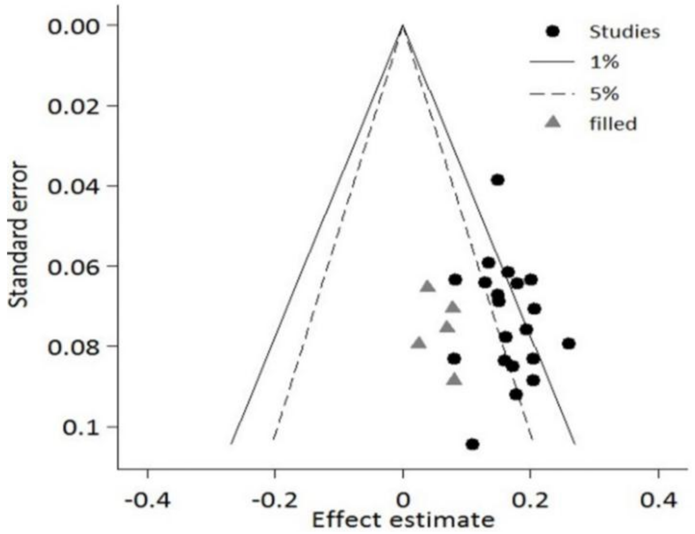

Figure S2. Funnel plot for *TAGAP* rs1738074 (A vs. G) with CD.

Table S1. Risk of bias assessment for genetic association studies of CD of studies included in the meta-analysis.

Domain and Item	Low Risk of Bias
Information bias	
Ascertainment of CD	
Clearly described objective criteria of diagnosis of CD	Yes
Not clearly identified	No
Did not mention	Unclear
Ascertainment of controls	
Controls were non-CD and without family history	No
Mentioned the sources of controls	Yes
Not described	Unclear
Ascertainment of genotyping examination	

Genotyping done under "blind" conditions of case specimens and control specimens	Yes
Genotyping of cases and controls was performed together	Yes
Genotyping error rate < 5%	Yes
Quality control procedure (e.g., reanalysis of random specimens, by using different genotyping methods for analysis, analysis if replicate sample)	Yes
Unblind	No
Genotyping error rate > 5%	No
Did not mention what was done	Unclear
Confounding bias	
Population stratification	
No difference in ethnic origin between cases and controls	Yes
Use of controls who were not related to cases with clearly identification	Yes
Use of some controls who came from the same family	No
No report of what was done	Unclear
Other confounding bias	
Controls for confounding variables (e.g., age, gender, or BMI) in analysis	Yes
Not controlled for confounding variables	No
Not mentioned	Unclear
Selective reporting (for replication studies)	
Reported results of all polymorphisms mentioned in the objectives, no significant or not	Yes
Reported results of only significant polymorphisms	No
HWE	
HWE in the control group	Yes
HWD in the control group	No
HWE not checked or mentioned	No

Table S2. MOOSE checklist: The association of *LPP* and *TAGAP* genes with CD risks: a meta-analysis.

	Criteria	Brief Description of How the Criteria were Handled in the Review
Reporting of background		
√	Problem definition	The epidemiology of celiac disease.<br>The conflict results of genetic risk with celiac disease in different population based studies.
√	Hypothesis statement	We propose there are associations between gene polymorphisms *LPP* rs1464510 and *TAGAP* rs1738074 and celiac disease.
√	Description of study outcomes	The pooled OR and 95% confidence interval
√	Type of exposure	Genetic markers
√	Type of study designs used	The population based genetic epidemiological observational studies of celiac disease.
√	Study population	The populations from the whole world are all is considered our analysis.
Reporting of search strategy should include		
√	Qualifications of searchers	Two reviewers independently went through all titles and abstracts of the identified studies.
√	Search strategy, including time period included in the synthesis and keywords	Time period: from inception of PubMed, Web of Science and Embase up to October 2016.<br>Search strategy:<br>(((*LPP* or 3q28 or rs1464510 or "lipoma preferred partner") or "lim domain containing preferred translocation protein") and celiac disease) and celiac disease) or ((*TAGAP* or 6p25 or rs1738074 or "T-cell activation GTPase activating protein") AND celiac disease).
√	Databases and registries searched	PubMed, Web of Science and Embase
√	Search software used, name and version, including special features	PubMed, was accessed from the National Library of Medicine (free), Web of Science is available on the website of Jinan University Library, Embase is purchased in internet.
√	Use of hand searching	We searched bibliographies of retrieved papers and those of previous reviewers on the subject were examined for further relevant studies.
√	List of citations located and those excluded, including justifications	Details of the literature search process are outlined in the flow chart. The citation list for excluded studies is available upon request.
√	Method of addressing articles published in languages other than English	We had a restriction on language; our searching was limited to English.
√	Method of handling abstracts and unpublished studies	We included proceedings papers and assessed them for eligibility according to our inclusion and exclusion criteria. Unpublished studies were excluded in our analysis.
√	Description of any contact with authors	It is applicable; we contact the authors when we needed.
Reporting of methods should include		
√	Description of relevance or appropriateness of studies assembled for assessing the hypothesis to be tested	Detailed inclusion and exclusion criteria are described in the Methods section.

√	Rationale for the selection and coding of data	Data extracted from each of the studies were relevant to the population characteristics name of first author, year of publication, region of study population, source of controls, genotype method, diagnostic criteria, the number of cases and controls, the risk allele frequency in cases and controls, and the Hard-Weinberg Equilibrium (HWE).
√	Assessment of confounding	Detailed inclusion is described in the Methods section.
√	Assessment of study quality, including blinding of quality assessors; stratification or regression on possible predictors of study results	Sensitivity analyses by several quality indicators such as study size, study objects' ethnic, and another influent factors, in the Methods section.
√	Assessment of heterogeneity	Heterogeneity of the studies was explored with I^2 statistic that provides the relative amount of variance of the summary effect due to the between-study heterogeneity, detailed inclusion is described in the Methods section.
√	Description of statistical methods in sufficient detail to be replicated	Description of methods of meta-analyses, sensitivity analyses, meta-regression and assessment of publication bias are detailed in the methods. We performed fixed effects and random effects meta-analysis with Stata (Ver. 12) and the Comprehensive Meta-Analysis software (Ver. 12).
√	Provision of appropriate Tables and graphics	Tables 1–4, Figures 1–3, Tables S1–S3, Figures S1 and S2
Reporting of results should include		
√	Graph summarizing individual study estimates and overall estimate	Figure 2 and Figure 3
√	Table giving descriptive information for each study included	Table 1
√	Results of sensitivity testing	Table S3
√	Indication of statistical uncertainty of findings	95% confidence intervals were presented with all summary estimates, I^2 values and results of sensitivity analyses.
Reporting of discussion should include		
√	Quantitative assessment of bias	The forest plot and Egg's regression.
√	Justification for exclusion	All studies were excluded based on the pre-defined inclusion criteria in methods section.
√	Assessment of quality of included studies	Brief discussion included in Methods section.
Reporting of conclusions should include		
√	Consideration of alternative explanations for observed results	Discussed in the context of the results.
√	Generalization of the conclusions	Discussed in the context of the results.
√	Guidelines for future research	We recommend analyses that would correct for regression dilution bias.
√	Disclosure of funding source	No separate funding was necessary for the undertaking of this systematic review.

Table S3. The sensitivity analysis of *LPP* rs1464510 and CD risk (A vs. C).

Excluded Study	Country	Pooled OR	95% CI	*p*	I^2 (%)	*p*-Value for I^2
Plaza-Izurieta et al. [7]	Spain	1.26	1.23–1.30	<0.001	29.86	0.126
Sperandeo et al. [31]	Italy	1.25	1.21–1.29	<0.001	26.38	0.141
Dubois et al. [8]	UK 1	1.25	1.21–1.29	<0.001	30.34	0.103
	UK 2	1.24	1.20–1.28	<0.001	19.77	0.213
	Finland 1	1.26	1.22–1.30	<0.001	33.20	0.080
	The Netherlands	1.26	1.23–1.30	<0.001	29.51	0.111
	Italy 1	1.26	1.22–1.30	<0.001	32.38	0.086
	USA	1.26	1.22–1.30	<0.001	32.98	0.082
	Hungary	1.26	1.22–1.30	<0.001	33.23	0.080
	Ireland	1.26	1.22–1.30	<0.001	33.30	0.080
	Poland	1.26	1.22–1.30	<0.001	31.88	0.090
	Spain	1.26	1.22–1.30	<0.001	33.20	0.080
	Italy 2	1.25	1.21–1.29	<0.001	23.60	0.170
	Finland 2	1.26	1.22–1.30	<0.001	32.32	0.087
Coenen et al. [30]	The Netherlands	1.27	1.23–1.31	<0.001	11.63	0.312
Romanos et al. [33]	Italy	1.26	1.22–1.30	<0.001	32.44	0.086
Hunt et al. [9]	UK	1.25	1.22–1.29	<0.001	31.34	0.095
	Ireland	1.26	1.22–1.30	<0.001	30.31	0.104
	The Netherlands	1.27	1.23–1.30	<0.001	22.92	0.177
Van Heel et al. [10]	UK	1.26	1.22–1.30	<0.001	32.98	0.082

© 2017 by the authors. Submitted for possible open access publication under the terms and conditions of the Creative Commons Attribution (CC-BY) license (http://creativecommons.org/licenses/by/4.0/).
